# Economic burden of multiple sclerosis on Kuwait health care system

**DOI:** 10.1371/journal.pone.0216646

**Published:** 2019-05-14

**Authors:** Maryam S. Alowayesh, Samar F. Ahmed, Jasem Al-Hashel, Raed Alroughani

**Affiliations:** 1 Department of Pharmacy Practice, School of Pharmacy, Kuwait University, Jabriya, Kuwait; 2 Department of Neurology, Ibn Sina Hospital, Sabah Medical Area, Kuwait; 3 Department of Neurology and Psychiatry, Minia University, Minia, Egypt; 4 Department of Medicine, Faculty of Medicine, Kuwait University, Jabriya, Kuwait; 5 Division of Neurology, Department of Medicine, Amiri Hospital, Sharq, Kuwait; Universita degli Studi di Napoli Federico II, ITALY

## Abstract

**Background:**

Multiple Sclerosis (MS) is a chronic neurological disease with heavy economic and social burdens resulting in significant disability.

**Objective:**

This study aims to (1) measure the cost of health resources utilization by MS patients and (2) to examine the difference in utilization and its attributed costs amongst patients who may have a different course of MS and expanded disability status scale (EDSS) scores.

**Methods:**

A cross-sectional study using Kuwait National MS registry was conducted to estimate the costs of utilization of resources from 2011 to 2015.

**Results:**

Between the period 2011–2015, 1344 MS patients were included in the registry. The average annual cost per MS patient has increased from $10,271 in 2011 to $17,296 in 2015. Utilization of disease-modifying therapies (DMTs) was the main driver of costs reaching 89.9% in 2015. Throughout the five-year period, the occurrence of relapses decreased from 21.8% to 12.2% (*p* <0.0001). During this same period, ambulatory relapse treatment increased by 5.8% while hospitalizations decreased by 2.6%. Patients with a moderate EDSS score (3.5–6) had the highest average cost (p<0.0001) compared to mild and severe EDSS scores.

**Conclusions:**

Multiple sclerosis has been a significant economic burden on the Kuwait healthcare system. DMTs are the main driver of cost.

## Introduction

Multiple Sclerosis (MS) is a chronic debilitating disease with heavy economic and social burdens resulting in severe disability and social dependence.[1] Because of the early onset of MS, it can often occur during the patient’s most productive working years, thus creating potentially large societal costs.[2] In addition to its burden on the patients and society, the entire healthcare system shares the financial burden of MS. [3] As a result of relapses and the progressive nature of MS, patients require repeated hospitalizations during disease exacerbations or worsening of their neurological disabilities. [4] In North-American and European studies, it has been reported that MS patients are more than twice as likely to be hospitalized or to consult a healthcare professional than patients without MS.[5–9]

When compared with the direct medical costs of other chronic conditions described in the literature, MS ranked second behind congestive heart failure. [10] According to several recent MS cost-of-illness studies, direct medical costs accounted for 64–77% of all costs with DMTs being the main driver of cost.[10,11]

In Kuwait, reported prevalence rate of MS was 85.05 per 100,000 persons in 2011.[12] Despite this high prevalence rate, the economic burden associated with the disease in Kuwait, as well as in the Middle East, is unknown. Detailed knowledge of the costs of an illness can provide the essential background that is necessary for policymakers to make informed decisions regarding which areas of disease treatment need to be addressed first and to subsequently set up and prioritize health-care policies and interventions.[13] Economic burden studies have become increasingly important under fast-changing healthcare systems.[3] Therefore, our aim was to measure the cost of health resources’ utilization by MS patients during the period 2011–2015 and to follow up by examining the differences in utilization and attributed costs amongst patients with different EDSS scores.

## Methods

### Patients and data collection

Ministry of Health (MOH) institutional review board has approved this study.

Written consent forms have been obtained from the participants. This cross-sectional study was conducted using data from Kuwait National MS Registry. Established in 2010, this registry accounts for nearly 95% of the MS patients diagnosed in Kuwait.[14] The registry includes the neurology tertiary hospital, other peripheral hospitals that have neurology units, and MS clinics. All patients were assessed by neurologists who are experienced in MS diagnosis using the revised 2010 McDonald diagnostic criteria.[15] Patients were classified either as clinically isolated syndrome (CIS), RR MS, progressive relapsing (PR) MS, secondary progressive (SP) MS, or primary progressive (PP) MS.[16] Patients included in the registry are seen at least twice per year during scheduled visits. A range of laboratory and radiological investigations are routinely ordered depending on the patient’s clinical status and what DMTs have been prescribed. Additionally, unscheduled visits and investigations due to relapses, adverse events, or other medical events are recorded in the registry. The institutional ethical committee approved the study and informed consent forms were obtained from all patients.

### Costing

Based on Trisolini and colleagues’ conceptual model for MS costs, this study collected only direct medical costs.[17] Five years of data were collected from 2011–2015. All unit costs were obtained from Ministry of Health personnel. They included inpatient hospital admissions, outpatient visits, laboratory and radiological investigations, and medications. In the Kuwaiti healthcare system, all Kuwaitis are entitled to free public hospital care and are entitled to free MS-related prescription medications. Therefore, all direct expenditure is paid by the Kuwaiti healthcare system. For those reasons, the costs in this study were measured only for Kuwaitis since non-Kuwaitis are not covered by the Kuwaiti healthcare system. Coverage of the costs for non-Kuwaitis is made through the patient’s helping fund or by the patients themselves. Unit costs of direct medical expenses are summarized in Table 1.

**Table 1:** Unit costs of direct medical expenses.

Resource	Cost in US dollars****
Diagnosis at entry and when needed*****	
CSF	523.2
EP	327.0
MRI Brain	621.3
MRI Whole spine	1046.4
MRI Thoracic	523.2
MRI Cervical	523.2
**Diagnosis Labs at entry**	
ANA	41.2
ENA	45.5
Vitamin B12	32.4
TSH	29.4
Serum ACE	47.1
Anti-NMO IgG test (10% of the sample)	164.8
Diagnosis Labs for DMTs (before start and during the treatment, according to routine schema)	
CBC	20.6
RFT	53.0
LFT	47.1
Urine Microscopy	11.8
Treatment (DMTs)*	
Teriflunomide	1831.2
Interferon beta—1a (powder)	1308.0
Interferon beta—1b	1504.2
Fingolimod	2746.8
Alemtuzumab**	29430, 49050
Interferon beta—1a (solution)	1275.3
Rituximab***	3924.0
Dimethyl fumarate	2092.8
Natalizumab	3106.5
Symptomatic treatment (for EDSS>3.5)	
Baclofen	39.2
Oxybutynin	52.3
Fampridine	719.4
Ambulatory treatment*****	
Methylprednisolone (3 days course)	359.7
Methylprednisolone (5 days course)	555.9
Hospital treatment*****	
Methylprednisolone (5 days course)	392.4
Plasmopheresis	9810.0
Hospitalization (per day)	712.9
Outpatient visits*****	
New patients	130.8
Follow-up patients	65.4

* price per month

** 5-day course costs $49050 or 3-day course costs $29430, given annually ($4087.5 per month cost for 5-day course; $2452.5 per month cost for 3-day course)

*** 6-months course ($654 per month cost)

**** the exchange rate was used as for 19.01.2017: 1 KD = $3.27 (https://www.xe.com/currencycharts/?from=KWD&to=USD&view=5Y)

*****These costs include personnel costs.

Abbreviations: CSF–Cerebrospinal Fluid, EP–Evoked potential, MRI–Magnetic Resonance Imaging, ANA—antinuclear antibody, ENA–extractable nuclear antigen, TSH–thyroid-stimulating hormone, ACE–angiotensin converting enzyme, NMO–Neuromyelitis optica, CBC–complete blood count, RFT–Renal function test, LFT–Liver Function Test, DMT–Disease Modifying Therapie, EDSS–Expanded Disability Status Scale, MethylPred–Methylprednisolone

### Statistical analysis

Direct medical annual costs were presented from the healthcare perspective. Disability was quantified using the EDSS.[18] Patients were stratified based on the severity of their disability into three groups based on their EDSS score: mild (EDSS 0–3), moderate (EDSS 3.5–5.5), and severe (EDSS 6–9).[19–20] To compare the EDSS groups, a Chi-square test was used for categorical variables (gender, birth country, MS course) and ANOVA (age, age of onset, and duration of disease) or Kruskal-Wallis test (number of relapses and for cost comparisons) for continuous variables. For the duration of disease comparison, a robust version of ANOVA (Welch test) was used as this variable did not fit the homogeneity of variance assumption. For summarizing costs in each year, it was presented as means and confidence intervals (CIs). When calculating CIs, the skewness of the distribution has to be considered. The level of statistical significance was set at P < .05.

Pairwise comparison between years performed using Dunn's (1964) [21] procedure with a Bonferroni correction for multiple comparisons (adjusted p-values are used) was used. Pairwise comparisons z-tests with Bonferroni correction for multiple comparisons were also used to compare the use of different DMTs between the years by type of intake (ex. IV, PO, SC, IM).

To investigate the relationship between total cost and EDSS scores, a set of linear regression models were constructed based on the data of the period 2011–2015. The EDSS independent variable was used as a continuous or an ordinal variable (mild, moderate, and severe groups). A logarithmic transformation of the total healthcare costs data was applied because its distribution is skewed.

A set of mixed-effect models with patients as random intercept and EDSS group (continuous or ordinal variable with three groups), gender, year, age along with age at MS onset, and disease duration as fixed effect were performed. An interaction term between the year and EDSS groups was added to the models. A final mixed-effect model was chosen based on the AICC criterion (Hurvich and Tsai [22]) and multi-collinearity between covariates.

## Results

There were 1344 patients included in the study who were recorded in the Kuwait national MS registry during the period 2011–2015 (Table 2). No patients were excluded for incomplete data. The majority were females (n = 896, 66.7%), and Kuwaiti national represented 87.6% (n = 1143) of the studied cohort. The mean age at MS onset was 26.8 (±8.8) years with a mean duration of disease of 8.7 (±6.9) years. Most patients had RR course (n = 990, 75.9%). Also, the majority of patients had a mild EDSS score (0–3) (n = 893, 77.2%). The relapse rate decreased significantly from 21.8% in 2011 to 12.2% in 2015; *p* <0.0001).

**Table 2:** Demographic and clinical characteristics of the participants.

Variables	Period	Total^a^	EDSS categories			p-value
			Mild (0–3)	Moderate (3.5–5.5)	Severe (6–9)	
Duration of the disease (years)	2011–2015	8.7 (6.9)	6.7 (5.2)	11.2 (5.8)	18 (7.6)	< .0005
Age of onset (years)		26.8 (8.8)	26.7 (8.7)	28.4 (9.6)	27.2 (9.1)	.146
Female, n (%)		865 (66.3)	616 (69.0)	67 (56.3)	83 (57.2)	.001
Birth country Kuwait, n (%)		1114 (87.8)	762 (87.5)	102 (86.4)	124 (89.9)	.670
Age (years)	2011	33 (10.1)	30.3 (9.6)	33.6 (10.8)	40.9 (11.6)	< .0005
	2012	33 (10.3)	30 (8.7)	31.5 (8.2)	40 (10.9)	< .0005
	2013	33.5 (10.4)	30.3 (8.8)	35.3 (10.1)	38.2 (10.2)	< .0005
	2014	34 (10.5)	31 (9)	36.8 (9.4)	40.2 (11.1)	< .0005
	2015	34.5 (10.6)	32.4 (9.5)	38.5 (10.2)	44.2 (10.6)	< .0005
EDSS, Mild (0–3), n (%)	2011	188 (71.2)				
EDSS, Moderate (3.5–5.5), n (%)		44 (16.7)				
EDSS, Severe (6–9), n (%)		32 (21.1)				
EDSS, Mild (0–3), n (%)	2012	341 (75.3)				
EDSS, Moderate (3.5–5.5), n (%)		58 (12.8)				
EDSS, Severe (6–9), n (%)		54 (11.9)				
EDSS, Mild (0–3), n (%)	2013	436 (80.4)				
EDSS, Moderate (3.5–5.5), n (%)		57 (10.5)				
EDSS, Severe (6–9), n (%)		49 (9.1)				
EDSS, Mild (0–3), n (%)	2014	558 (80.6)				
EDSS, Moderate (3.5–5.5), n (%)		60 (8.7)				
EDSS, Severe (6–9), n (%)		74 (10.7)				
EDSS, Mild (0–3), n (%)	2015	589 (83.3)				
EDSS, Moderate (3.5–5.5), n (%)		50 (7.1)				
EDSS, Severe (6–9), n (%)		68 (9.6)				
MS course, CIS, n (%)	2011–2015	135 (10.5)	111 (12.7)	5 (4.3)	0 (0)	< .0005^b^
MS course, PP, n (%)		35 (2.7)	9 (1)	8 (6.9)	10 (7)	
MS course, PR, n (%)		3 (0.2)	1 (0.1)	1 (0.9)	1 (0.7)	
MS course, RR, n (%)		990 (77.1)	756 (86.2)	91 (78.4)	35 (24.5)	
MS course, SP, n (%)		121 (9.4)	0 (0)	11 (9.5)	97 (67.8)	
Number of relapses	2011–2015	0.13 (.4)	0.15 (.4)	0.11 (.3)	0.04 (.2)	.005
No relapses, n (%)	2011–2015	1146 (87.8)	775 (86.8)	107 (89.9)	139 (95.9)	
1 relapse, n (%)		142 (10.9)	102 (11.4)	11 (9.2)	6 (4.1)	
2 relapses, n (%)		17 (1.3)	16 (1.8)	1 (0.8)	0 (0)	

Data are expressed as mean (SD) if not stated.

EDSS: Expanded Disability Status Scale; MS: multiple sclerosis; SD: standard deviation

MS course: CIS: Clinically isolated syndrome, PP: Primary progressive, PR: Progressive relapsing, RR: Relapsing-remitting, SP: Secondary progressive

^a^ The shares are calculated from the number of patients with available EDSS information.

^b^ p-value for MS course by EDSS group comparison may be invalid as there were many cells with low count

**Fig 1:**
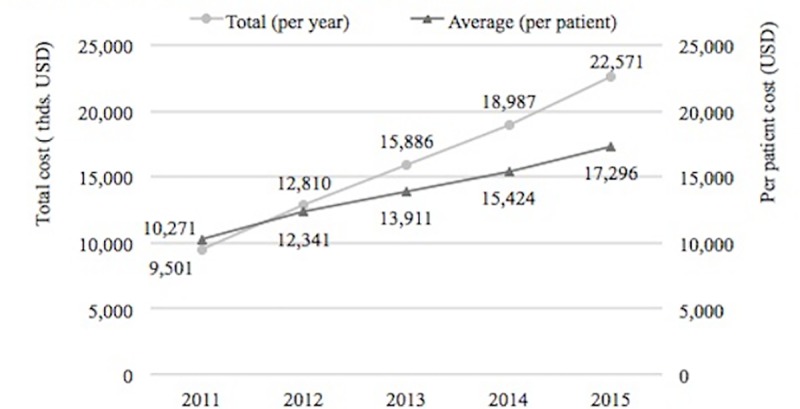
Total cost per year and mean cost per patient per year.

The total direct medical costs of MS increased significantly (*p* < .005) from year 2011 to 2015 as shown in Fig 1. There was also a significant difference between per-year and per-patient mean values between 2011 and 2012 years (p = .016) followed by significant growth with a one-year lag: 2013 being higher than 2011, 2014 higher than both 2011 and 2012, and 2015 being higher than 2013 and all earlier years (all p < .005). This can be interpreted as a significant trend towards an increase in mean per-patient costs during the 2011–2015 period. Detailed mean per-patient costs have been summarized in Table 3.

**Table 3:** Detailed mean costs per patient by year (in USD).

	2011		2012		2013		2014		2015	
	Mean	95% CI	Mean	95% CI	Mean	95% CI	Mean	95% CI	Mean	95% CI
Total cost	10271	(9543–11000)	12341	(11553–13130)	13911	(13095–14727)	15424	(14588–16260)	17296	(16464–18127)
Diagnosis (CSF + EP + MRI + Diagnosis at entry)	246	(215–275)	338	(307–374)	462	(426–497)	429	(399–459)	379	(354–405)
Diagnosis (DMTs laboratory tests)	130	(120–139)	157	(147–168)	180	(169–191)	211	(200–223)	233	(222–245)
Treatment (DMTs)	8639	(7945–9296)	10450	(9791–11217)	12166	(11414–12933)	13442	(12651–14222)	15538	(14773–16357)
Symptomatic treatment (EDSS)	800	(635–969)	1050	(880–1230)	903	(740–1069)	1059	(891–1237)	880	(743–1031)
Hospital treatment	401	(245–572)	256	(139–391)	64	(18–127)	118	(34–243)	124	(54–211)
Ambulatory treatment	17	(12–24)	23	(17–30)	53	(44–62)	66	(56–76)	50	(42–60)
Outpatient visits	39	(35–44)	68	(63–73)	83	(77–89)	98	(93–104)	91	(86–96)

CSF: Cerebrospinal fluid; EP–Evoked potential; MRI: Magnetic resonance imaging

DMT: N,N-Dimethyltryptamine; EDSS: Expanded Disability Status Scale

The main driver of costs were DMTs since their overall share of the total cost has increased significantly from 2011 to 2015 (84.1% to 89.8% respectively; *p* <0.0001). Excluding hospitalization costs, all other shares of costs remained the same during the years and its (hospitalization) share in total cost decreased significantly from 2011–2015 (3.9% to 0.7% respectively; *p* <0.0001). Detailed resource utilization for each year was summarized in Table 4.

**Table 4:** MS resource utilization by year, n (proportion of patients, %).

Year	2011	2012	2013	2014	2015	p-value
Pairwise comparisons*	A	B	C	D	E	
Total Diagnosis	290 (31.4)	378 (36.4)	540 (47.3)	642 (52.2)	640 (49.0)	< .0005
			AB	AB	AB	
CSF analysis	14 (1.5)	22 (2.1)	18 (1.6)	24 (1.9)	10 (0.8)	0.069
EP	11 (1.2)	19 (1.8)	11 (1.0)	18 (1.5)	4 (0.3)	.007
		E		E		
MRI	243 (26.3)	344 (33.1)	497 (43.5)	617 (50.1)	630 (48.3)	< .0005
		A	AB	ABC	AB	
Diagnosis at entry	127 (13.7)	123 (11.8)	137 (12.0)	121 (9.8)	98 (7.5)	< .0005
	DE	E	E			
Diagnosis (Labs for DMTs)	467 (50.5)	566 (54.5)	642 (56.2)	723 (58.7)	828 (63.4)	< .0005
				A	ABC	
Treatment (DMTs)	478 (51.7)	575 (55.4)	657 (57.5)	735 (59.7)	850 (65.1)	< .0005
				A	ABCD	
Symptomatic treatment (EDSS)	76 (8.2)	112 (10.8)	106 (9.3)	134 (10.9)	118 (9.0)	0.159
Hospital treatment	33 (3.6)	20 (1.9)	7 (0.6)	8 (0.6)	13 (1)	< .0005
	CDE					
Ambulatory treatment	31 (3.4)	44 (4.2)	115 (10.1)	148 (12.0)	120 (9.2)	< .0005
			AB	AB	AB	
Outpatient visits	264 (28.5)	453 (43.6)	542 (47.5)	692 (56.2)	706 (54.1)	< .0005
		A	A	ABC	ABC	

CSF: Cerebrospinal fluid; EP–Evoked potential; MRI: Magnetic resonance imaging; DMT: N,N-Dimethyltryptamine; EDSS: Expanded Disability Status Scale

* Results are based on two-sided tests with significance level .05. For each significant pair, the key of the category with the smaller column proportion appears under the category with the larger column proportion

**Fig 2:**
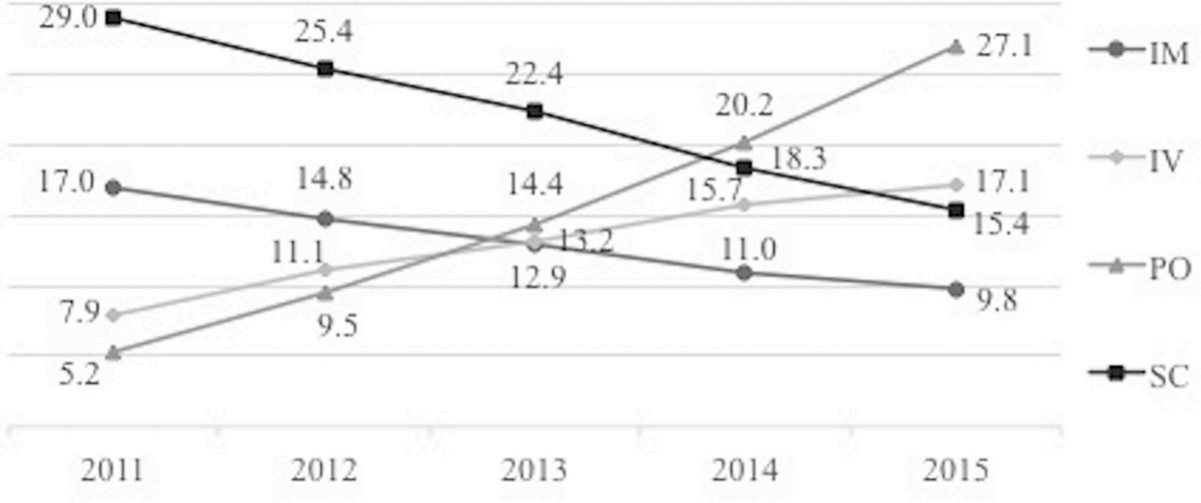
Utilization of treatments (DMTs) 2011–2015, by type of intake* (%). * IM (intramuscular): Avonex; IV (intravenous): Tysabri, Lemtrada, Rituxan; PO (per os): Aubagio, Tecfidera, Gilenya, SC (subcutaneous): Betaferon, Rebif.

There was a statistically significant (p < .05) growth in intravenous (IV) and oral (PO) DMT utilization as shown in Fig 2. The share of IV and PO DMTs usage was significantly higher in 2015 compared to 2011 (p < .05) and all intermediate shares lie within the 2011 and 2015’ values. The shares of intramuscular (IM) and subcutaneous (SC) utilization decreased measurably within the 2011–2015 period (the 2011 and 2015 shares differ significantly (p < .05) and all intermediate values lie within these borders).

Table 5 showed the results of the final regression model selected based on AICC and collinearity criteria (age of onset and interaction between EDSS and years were removed due to collinearity with other covariates). According to these results and after adjustments for confounding factors, patients with mild EDSS level (0–3) had 41% significantly lower costs compared to the severe group. Patients with moderate EDSS level (3.5–5.5) had 31% higher costs compared to the severe group (6–9). The distribution of total costs was not significantly different between males and females (ratio = 1.13; p-value = 0.159). Total costs did increase significantly when disease duration increased (5% increase in total cost for 1-year increase in disease duration) and across years (55%, 34%, 20%, and 17% lower cost for 2011, 2012, 2013, and 2014 compared to 2015). However, total costs decreased noticeably when age increased (2% decrease in total cost for 1-year increase in age).

**Table 5:** Mixed-effect model results for total cost in the period 2011–2015 by EDSS categories.

Parameter	Ratio*	95% Confidence Interval		P-value
		Lower Bound	Upper Bound	
Intercept	21474,08	14307,48	32230,42	p < .001
Mild EDSS^a^	0,59	0,46	0,76	p < .001
Moderate EDSS^a^	1,31	1,01	1,71	0,043
Male^b^	1,13	0,95	1,33	0,159
MS Length in years	1,05	1,03	1,06	p < .001
Year = 2011^c^	0,45	0,38	0,52	p < .001
Year = 2012^c^	0,64	0,56	0,72	p < .001
Year = 2013^c^	0,80	0,72	0,90	p < .001
Year = 2014^c^	0,83	0,74	0,92	p < .001
Age in years	0,98	0,97	0,99	p < .001

* Ratio = exp (estimate)

^a^ Severe EDSS is reference group;

^b^ Female is reference group; Year = 2015 is reference group

Analysis of estimated marginal means revealed that the average total cost per patient per year was $16,848 for moderate EDSS group, $12,849 for severe EDSS group, and $7,593 for mild EDSS group (data are not shown in a table).

## Discussion

Directed towards the healthcare payers, this study provides an insight into the distribution of costs and the resource utilization that is required across direct costs categories and patients with varying levels of disability. The economic impact of increasing disability (increase in EDSS score) is particularly obvious in the increase in direct costs between mild and moderate disability. Mean annual direct medical costs increased from $7,593 to $16,848 per person with mild and moderate disease, respectively. This increase did not apply to the severe disease category; their annual mean direct medical cost was $12,849. This was in direct contrast to other studies that found that severe disease incurred the highest costs.[11,23] The difference in statistical results can be explained by the fact that the other studies also captured indirect costs like early retirement and productivity loss along with direct non-medical costs associated with disability such as caregiver costs, physiotherapy costs, and transportation. This particular study did not include those additional categories.

The average annual direct medical cost per MS patient has increased from $10,271 in 2011 to $17,296 in 2015. These figures were similar to other studies that looked at direct costs only.[3,10] The average annual cost per MS patient in 2015 was $17,296; this cost is similar to what other countries are spending on MS.[3,10]

A large multi-national cost-of-illness of MS study, done in 16 European countries, was published in 2017.[24] It confirmed that an increase in disability is directly related to an increase in cost.[24] Additionally, it confirmed that cost in severely disabled patients is not driven mainly by direct medical costs. Medical costs only accounted for 26% of the overall cost category. Instead, the rise in costs was primarily a result of the loss of productivity and an accompanying decline in quality of life for those with advancing disability.[24] Productivity decreased from 82% in mildly disabled patients to 8% in severely disabled patients.[24] While utility decreased to less than zero in severely disabled patients, fatigue and cognitive difficulties produced a significant impact on utility.[24] A recent study done in the US also found that the percentages of cost increased alongside increased disability. [25] Annual costs per patient were $51,825, $57,889, and $67,116 for mild, moderate, and severe disability, respectively.[25] It is worth noting that the costs of healthcare in the US are significantly higher than other parts of the world. However, this may be due to the way healthcare is managed and financed within the US.

A recently published systemic review of MS cost-of-illness studies done in OECD (Organization for Economic Co-operation and Development) countries (18 European countries plus the US and Canada) showed that bottom-up costing approach and prevalence approaches were most common, which is the same approach used in this study.[26] Also, it reported that the cost ratios between different severity levels within studies were fairly stable, to the ratio of 1 to 2 to 3 for disability level categories.[26] Further, it mentioned that drugs were the main cost drivers for MS-patients with low disease severity, while the main cost components for groups with more advanced MS symptoms were production losses due to MS and informal care, all of which are similar to the results of our study. [26]

Relapse rate has halved from 2011–2015 (21.8% to 12.2%). This suggests that compliance and possibly efficacy were better with new-generation DMTs.[27–30] Although comparisons across clinical trials is challenging, especially in the absence of head-to-head comparison trials, several retrospective propensity-matched studies showed that new generation DMTs (specifically natalizumab, fingolimod, and alemtuzumab) were associated with a lower risk of relapse compared to the platform therapies (Beta interferon and Glatiramer Acetate) [27–30] Recent data has shown that dimethyl fumarate has a similar efficacy to fingolimod.[31–32] It would be better to state that in the last decade, high efficacy DMTs have emerged with lower relapse rates and MRI activities.

It is also important to note that the establishment of multi-disciplinary MS clinics and the referral to MS specialists resulted in more patients being escalated or switched to high efficacy DMTs and these actions improved the overall adherence rates as per the neurologists’ observation. Both of these factors may have impacted, in a positive way, on the relapse rate in the last few years. In this five-year period, more relapses were treated in ambulatory clinics (85.2% in 2011–92.6% in 2015) and less needed hospitalizations (14.8% in 2011–7.4% in 2015). Treating patients’ relapses in an ambulatory MS clinic costs much less than treating them in a hospital. A relapse that is treated in an ambulatory MS clinic costs around $359–555 per patient, $359 if it was a 3-day course of methylprednisolone, and $555 if it was a 5-day course of methylprednisolone. By contrast, an in-hospital treatment of a relapse would cost around $2,371 if it was 3-day admission and $3,919 if it was 5-day admission.

One US study looked at the excess costs that a patient with relapse will have during the year. It compared patients with relapse and patient with no relapse.[33] Patients with relapse were grouped into a low/moderate severity group and the other was the high severity group.[33] The low/moderate severity group and high severity group incurred an excess of $8269 and $24,180 in direct costs compared to the no-relapse group respectively.[33] Another study done in Ireland looked at the direct and indirect cost of an MS relapse.[34] The directs costs of a patient relapse ranged from $469 (low-intensity relapse) to $6,353 (high-intensity relapse). The significant difference between the two sets of costs was mainly caused by hospital admission costs in the high-intensity relapse group, an amount that produced almost 75% of the cost. [34]

From 2011 to 2015, oral DMTs utilization has increased from 5.2% to 27.1% since most of the newer DMTs were oral and more convenient for patient usage. Subsequently, several observational studies showed improvements in adherence with orals compared to injectables. [35–37] Highly efficacious drugs with greater adherence rates provide the greatest real-world effectiveness and may offer the best economic value.[38] However, highly efficacious therapies with low adherence may yield real-world efficacy that is considerably lower than that observed in strictly monitored clinical trials.[38] Moreover, a US study explored the effect of adherence to DMT on the overall spending on MS.[39] It found that adherence to DMT notably reduced the possibility of relapse by 42%, hospitalization by 52%, and emergency visits by 38% (all, P<0.0001). [39] Adherent patients would be predicted to have on average 0.7 fewer outpatient visits each year versus non-adherent patients (P<0.0001). Based on the differences in predicted mean costs, adherence (vs non-adherence) would decrease the total annual medical care costs by $5,816 per patient, including hospitalization costs by $1,953, emergency visits by $171, and outpatient visits by $2,802. [39]

## Limitations

This study only examined the direct medical costs of MS. Looking at both direct and indirect costs of MS would give a more comprehensive picture of the burden of the disease. A future study is planned to survey patients and explore indirect costs, the patients’ quality of life, and the patients’ adherence to newer DMTs. Moreover, for this particular study, some unit costs of laboratory procedures were hard to obtain since the Ministry of Health (MOH) only had the cost of the machine and reagent. To overcome that limitation, laboratory personnel were consulted to give an estimate of some of these laboratory procedures. In addition, micro-costing an MS hospitalization was challenging. As a result, the approach that was used was to get the estimate from MOH for a neurology-related hospital admission and then add the cost of specific MS drugs and diagnostics.

This study is a cross-sectional study, which means that it is estimating the cost at this point in time. This time-related factor may bias some of the results since patients may be switched from one DMT to another within a short period due to disease reactivity. Additionally, given the referral bias to MS clinics, our findings cannot be generalized as the main source of cost was driven by the DMT prescriptions that are dependent on the treatment protocol used in our center.

## Conclusion

Multiple sclerosis continues to be a significant economic burden on the Kuwait healthcare system. Disease-modifying therapies seem to be the main driver of cost. Over recent years, oral and infusion therapies (new-generation DMTs) have been prescribed more often and, as a result, the overall relapse rates have decreased.
